# Design of a Multifunctional Resin-Based Outdoor Spherical Robot Shell for Ultrahigh Visible to Near-Infrared Transmittance and Mid-Infrared Radiative Cooling

**DOI:** 10.1021/acsomega.4c09954

**Published:** 2025-01-11

**Authors:** Wei-Lin Wu, Shang Yu Tsai, Yu-Chieh Lo, Hsueh-Cheng Wang, Hsuen-Li Chen, Dehui Wan, Fu-Hsiang Ko

**Affiliations:** † Department of Materials Science and Engineering, National Yang Ming Chiao Tung University, No. 1001, University Road, Hsinchu 30010, Taiwan; ‡ Department of Electronics and Electrical Engineering, National Yang Ming Chiao Tung University, No. 1001, University Road, Hsinchu 30010, Taiwan; § Department of Materials Science and Engineering, National Taiwan University, No. 1, Sec. 4, Roosevelt Road, Taipei 10617, Taiwan; ∥ Institute of Biomedical Engineering, National Tsing Hua University, No.101, Sec. 2, Kuang Fu Road, Hsinchu 30013, Taiwan

## Abstract

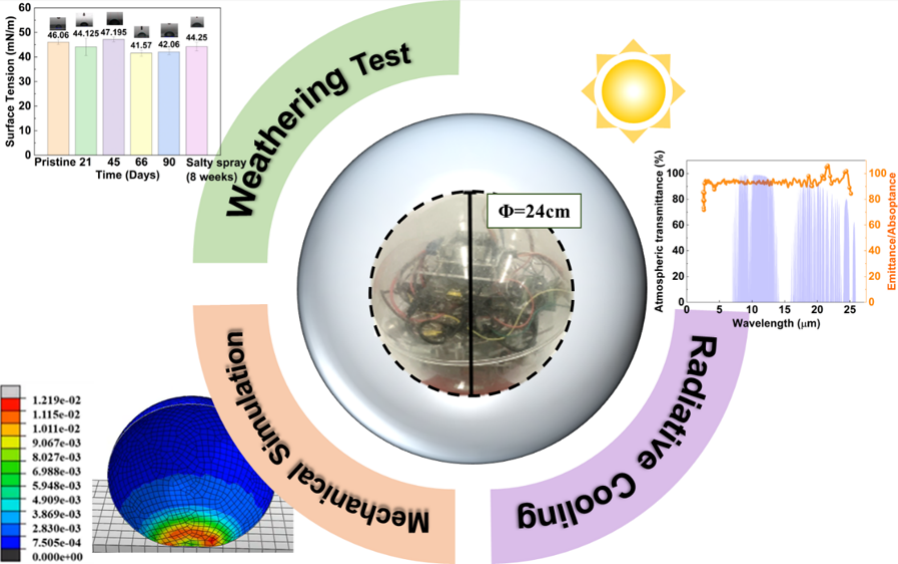

As robots undertake increasingly complex tasks, such as real-time visible image sensing, environmental analysis, and weather monitoring under harsh conditions, design of an appropriate robot shell has become crucial to ensure the reliability of internal electronic components. Several key factors, such as the cooling efficiency, visible transparency, mechanical performance, and weathering resistance of the shell material, are proposed in this research to ensure future robot functionality. In this study, a polymeric double-layered shell for fabrication by stereolithography 3D printing was designed, featuring a porous outer layer and a spherical inner shell. The inner spherical shell provides approximately 90% transmission in the visible to near-infrared wavelength range (450–1050 nm) and ensures the proper functioning of the optical devices, such as cameras, lidar, and solar cells, inside the robot. In addition, the inner shell material displays high emittance in the mid-infrared range (5–20 μm) to facilitate effective radiative cooling and protect the robot control system from thermal damage. The 3D-printed inner shell is exposed to a real environment for three months, and its stable optical and mechanical performance confirms its weather resistance ability. Moreover, the 3D-printed outer robot shell promotes mechanical strength while the robot is moving. The optimal 50% porous outer shell is designed to protect the inner shell from continuous moving impact. Finite element simulations are also used to show that the 50% porosity of the outer shell significantly reduces the strain energy upon impact. Compared with a conventional single-layer design with a strain energy of 130 mJ, the double-layered shell with 50% porosity exhibits a reduced strain energy of 22.09 mJ. This double-layered design, which offers excellent weather resistance, high visible transparency, and effective radiative cooling, is promising for future applications in both land and water robot shells.

## 1 Introduction

**Figure 1:**
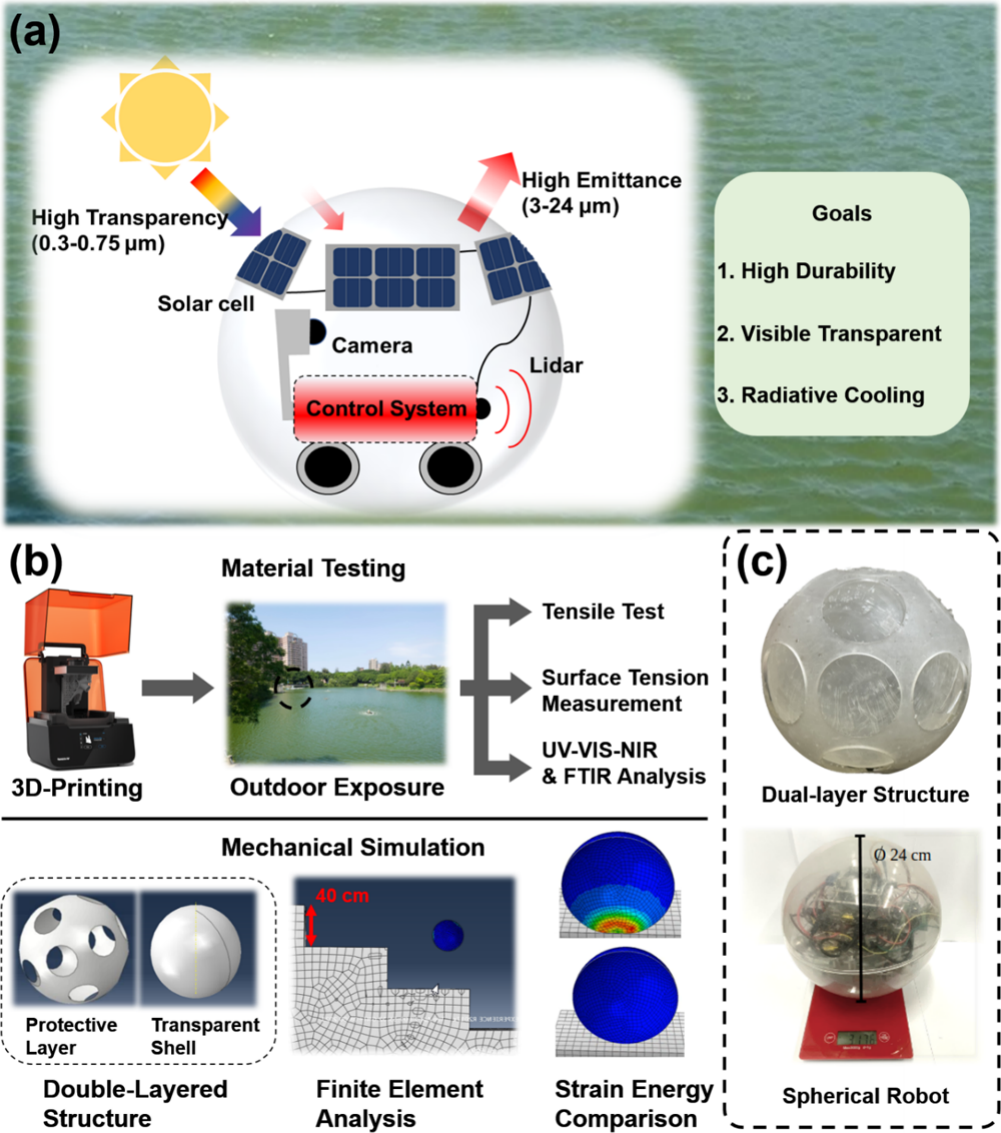
(a) Schematic illustration of the structural design of a multifunctional rolling robot for an internal electronic component. (b) Experimental setup for material testing of 3D-printed samples and mechanical impact simulation of a double-layered structure. (c) Actual photograph of the fabricated sample and car-driven spherical robot.

The application of robots reduces the risks associated with various human tasks while providing reliable visual detection and image analysis. In fields such as maritime inspection,^1^ agricultural sensing^2^ and resource exploration,^3^ direct human involvement is no longer suitable because of the requirement of extensive working hours or exposure to extreme environments. With the rapid development of unmanned technology, significant attention has been directed to the improvement of various robots, particularly autonomous amphibious robots, which have improved in recent research.^4^ Amphibious robots, which show excellent performance on both land and water, are widely used for outdoor tasks and monitoring applications in extreme environments.^5^ Among several shape designs, spherical robots have advantages such as rolling capability, symmetricity, large internal space, and flexible placement of electronic sensors.^6^ Furthermore, the symmetric shell enhances good radiation isotropy, which promotes radiative cooling.^7^ For outdoor tasks, the internal heat buildup of the robot caused by power consumption is a primary concern, which can be effectively addressed through radiative cooling. In addition to cooling efficiency, visible transparency is essential for the proper functioning of internal image sensors. Furthermore, mechanical strength and weathering resistance are critical characteristics for ensuring the long-term reliability of internal electronic devices. However, despite advances in robot design, research on robot shells is still limited. Robot shells are usually fabricated of aluminum or PMMA materials. However, while sturdy, the aluminum frames are heavy and opaque. Meanwhile, traditional extruded PMMA shells lack mechanical strength. As shown in Figure 1a, the aim of this study is to explore the radiative cooling performance, visual transmission, and mechanical strength properties of robot shells during movement. In particular, this study uses 3D printing technology to increase the mechanical strength and achieve more customized shape designs of robot shells.

The use of three-dimensional (3D) printing technology for the fabrication of various robots has gained widespread acceptance^8^ because this method is relatively inexpensive and eliminates the need for extra molds. The 3D printing market has experienced an annual growth rate of 10% in recent years, and this trend is expected to continue owing to its advantages.^9^ The main advantage of 3D printing is its ability to directly convert CAD designs into physical samples, allowing designers to concentrate on the design process instead of managing tedious fabrication processes. 3D printing methods include digital light processing (DLP), fused deposition modeling (FDM), and stereolithography (SLA).^10^ The SLA method, which utilizes photopolymerization, can obtain a finer interlayer thickness than FDM, which relies on thermal melting. Furthermore, SLA achieves higher resolutions than DLP due to its point-by-point solidification process.^10,11^ In this study, the SLA technique was used to print mechanical test samples and thin films for optical testing to evaluate material properties. The samples also underwent long-term outdoor exposure to evaluate the weathering resistance of the printed products.

In addition to physical properties, thermal management is another important consideration for robot shells due to the need to ensure the proper functioning of internal electronic sensors.^12^ Recently, passive daytime radiative cooling (PDRC) has become a high-profile topic because of the worsening of Earth’s climate change.^13^ An increasing research effort has been devoted to the investigations and development of novel materials and fabrication methods in this field.^14^ Compared with conventional cooling methods, such as air conditioning and water cooling, radiative cooling is a passive method for transporting heat away from a heat source without consuming energy. Since radiative heat dissipates into outer space (which has a temperature of ∼3 K), it passes through the first atmospheric transparency window (8–13 μm) and the second atmospheric transparency window (16–25 μm).^15^ This approach requires a suitable cooling material with high emissivity in the aforementioned atmospheric windows. In the field of mechanical engineering, the finite element method (FEM) is considered a powerful tool for solving the problems of interactions and contact sites between parts. Finite element technology is time-efficient in terms of labor, energy and material resources^16^ that has demonstrated its applicability for long-term aging and repeatable crash tests. Commercial finite element analysis (FEA) software (namely, Abaqus) has been widely used in microelectronics,^17^ mechanical simulations^18^ and architectural applications.^19^

In this study, the specific design shown in Figure 1b for a spherical robot shell fabricated from a 3D-printed resin was proposed because it provides protection for the sensor using a porous outer layer and an inner layer. The proposed structural material offers the advantages of visible light to near-infrared transmission, mid-infrared radiative cooling ability and relatively high mechanical strength for future robot applications. By using a dual-layer porous shell structure, a shell with a specific impact resistance retains the optical properties of high transparency under visible light and high emissivity in the atmospheric window. The tensile strength of the printed robot material was measured, and standard salt spray corrosion tests and surface energy characterization were performed to determine its mechanical properties and outdoor weathering resistance characteristics.

## 2 Experimental Procedures

### 2.1 Resin Material and 3D Printing Shell Durability

The two types of polymeric materials displayed in Table S1 were used, both of these materials were photopolymer resins. An inner shell material (i.e., clear resin) offered good transparency in the visible region for monitoring purposes while moving. In addition, an outer shell material (i.e., durable resin) was designed for long-term flexibility and durability. The inner clear resin used for 3D printing consisted of a mixture of 55–75% urethane dimethacrylate (UDMA), 15–25% methacrylate monomer, and <0.9% diphenyl(2,4,6-trimethylbenzoyl) phosphine oxide (photoinitiator). The protective layer (outer layer resin) used for 3D printing was also obtained from Formlabs Co. and contained a mixture of 45–65% UDMA, 15–25% methacrylate monomer, 10–20% acrylate monomer, and <1.5% diphenyl(2,4,6-trimethylbenzoyl) phosphine oxide (photoinitiator).

3D printing techniques have played an important role in engineering and laboratory-scale experiments. Tensile tests and optical measurements were conducted on two design samples, i.e., a dog bone-shaped sample (as shown in Figure S1) with a neck with the cross-sectional dimensions of 2 × 1 mm and a film sample with the dimensions of 1 cm × 1 cm × 1 mm. Both the dog bone-shaped samples and film samples were designed in AutoCAD and printed using a commercial SLA 3D printer (Form 3, Formlabs Co., Somerville, MA, USA). All the printed samples were cured at 60 °C for 20 min using a 405 nm light-emitting diode (LED) lamp at an output power of 1.25 mW/cm^2^ to maintain the mechanical strength of the printed objects. A mechanical impact simulation was also conducted on a typical rolling robot model, as shown in Figure 1c. The inner shell has a diameter of 24 cm, with a surface area of approximately 1808.64 cm^2^. The outer protective shell has a slightly larger diameter of 24.2 cm. For the 50% porous structure, each circular pore has a diameter of 7.6 cm, corresponding to an area of approximately 90.4 cm^2^ per pore. Both shells have a thickness of 2 mm, and the total weight of the robot, including the internal electronics, is 3.2 kg. To minimize the dispersion of visible light, the inner shell undergoes a polishing procedure to increase its smoothness and clarity.

A two-month standard accelerated corrosion test of the above printing materials was conducted to evaluate the long-term reliability of the robot shell material. The cyclic corrosion test (CCT) was carried out according to Severity 7 of IEC 61701:2011, which is similar to the Mercedes–Benz corrosion test applied in vehicle resistance tests. One test cycle had a duration of 168 h and consisted of four salt spray periods, each lasting 2 h. Each salt spray period was followed by storage in humid conditions for 22 h. After humidity storage, each sample experienced one storage period of 3 d under a standard atmosphere. The total test duration for the printed shell material was 8 weeks, and the shell material was subjected to tensile tests and surface energy measurements. This corrosion test was beneficial for evaluating the long-term reliability of robot shell materials with respect to impacts during movement.

### 2.2 Weathering Evaluation and Characterization

The printed shell materials were immersed in a lake at our university to verify the durability of the robot shell candidate material in a real outdoor environment. The weather resistance of the printable resin was then evaluated by measuring the changes in the surface tension, tensile strength, and ultraviolet–visible (UV–Vis) and Fourier transform infrared (FTIR) spectra of the material. A double-layered structure was introduced and simulated by performing a finite element simulation of a powerful crack^20^ to evaluate impact resistance. The procedure for three-month lake immersion of the printed resin is described below. First, the printed dog bones samples and film samples were placed in a bamboo lake at the National Yang Ming Chiao Tung University. Periodically, the samples were brought back to the laboratory and subjected to regular tensile strength and surface energy measurements. For the tensile strength, each dog bone-shaped sample was stretched by a dynamometer (FG-6020SD, Lutron), and the maximum tensile strength of the sample at the breaking point was recorded. Furthermore, the surface tension was calculated using a contact angle meter (SURFTENS OEG, Germany) with two solvents (deionized water and diiodomethane) of different polarities. A UV–visible spectrophotometer (UV-2600i, Shimadzu) with an integrating sphere was used to measure the UV–vis-near-infrared (IR) spectrum. Finally, the emissivity values in the mid-IR region at the wavelengths between 5 and 20 μm, and particularly in the atmospheric window, were analyzed using an FTIR spectrometer (Vertex 70, Bruker).

### 2.3 Simulation and Real Stair Drop

In this study, the built digital models and combined parts were examined using the Abaqus FEA software. Then, simulation results were collected for each element based on the material properties, boundary conditions and contact conditions. Drop tests with single-layer, dual-layer and porous structure robot shells were proposed to achieve an effective understanding of the mechanical performance. The dual layer was divided into an outer protective layer and an inner transparent layer. In the optimization of the porous structure, circular shapes offer higher symmetry and distribute strain more evenly than other common designs, such as square holes and linear structures. This approach not only reduces material consumption but also ensures that the optical properties of the inner layer are maintained. To increase the simulation accuracy, accurate measurements of meshing^21^ and definitions of boundary conditions, material strengths, and contact characteristics are essential. First, the relationship between the residual stress and mesh size is analyzed. The properties of each material are summarized in Table S1. In the field simulation, the rolling robot rolls down four steps to a height of 50 cm, for a total height of 2 m. Then, an assessment is conducted to determine if any mesh has been lost, which could indicate damage to the ball shell. On this basis, the impact state of the shell is quantified from an energy perspective. After conducting the stair drop simulation with a single-layer robot shell, another simulation is performed by changing the shell structure and the material used for printing the robot shell to achieve good impact resistance.

## 3 Results and Discussion

### 3.1 Mechanical Strengths of Printed Robot Shell Materials for Weathering Resistance

**Figure 2:**
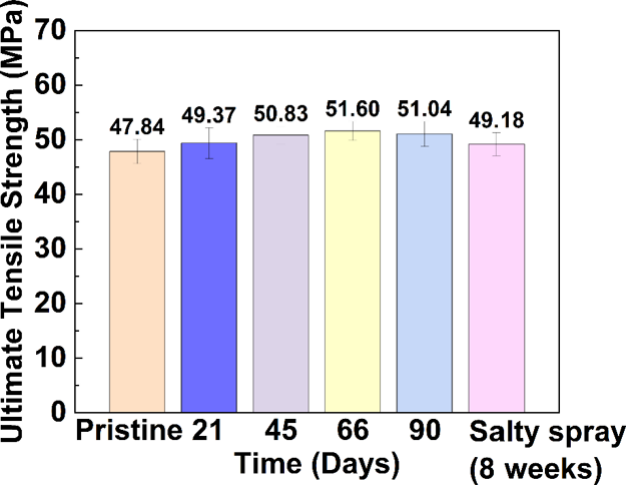
Ultimate tensile strengths of the printed robot shell materials in the bamboo lake following three months of outdoor exposure (*n* = 5).

Polymeric materials gradually degrade under exposure to corrosive environmental conditions such as ambient temperature, sunlight irradiation, oxygen adsorption, and humidity. This degradation process involves changes in the mechanical strength due to the reorganization of functional groups,^22^ which leads to the malfunction of the inner device of the robot. The tensile properties of the dog bone-shaped samples were measured at different exposure times to evaluate the degradation effect. The ultimate tensile strengths of the printed samples immersed in lake water for three months are depicted in Figure 2. The pristine samples (*n* = 5) have a mean tensile strength of 47.84 MPa, with a standard deviation of 2.23 MPa. After 3 weeks of outdoor exposure, the tensile strength increased slightly to 49.37 ± 2.82 MPa. The tensile strength gradually increased to 50.83 ± 1.62 and 51.60 ± 1.74 MPa after 45 and 66 d of lake water immersion, respectively. However, after three months of immersion, a slight reduction in the tensile strength to 51.40 ± 2.30 MPa was observed. Although the average tensile strength decreases by 0.2 MPa, the standard deviation indicates that the measured results are very close to each other among the robot shell material samples immersed for different numbers of days.

The variation in the ultimate tensile strength of the material with increasing immersion time is related to the sunlight-based photodegradation effect. Some researchers^23^ have suggested that this increase can be attributed to the oxidation of the polymeric material, which causes chain scission, molecular reorganization and/or additional cross-linking. However, a statistical analysis of the tensile strength indicated a lack of significant difference (*p* value > 0.05). Therefore, the variation in the tensile strength values may be attributed to the differences between the 3D-printed robot shell materials of the individual samples (*n* = 5).^24^ This behavior can be attributed to the photocuring agent remaining in the resin after printing. The limited amount of residual agent enhances the strength of the robot shell material after immersion in the lake for various durations. The small change in the tensile strength due to the residual curing agents in 3D-printed resin can be improved by posttreatment with UV curing. Moreover, small changes have no effect on the stability of the resin used in robot shells. Thus, the similar values of the tensile strength obtained for various samples confirm the mechanical stability of the robot shell materials.

Figure 2 shows that the tensile strength resulting from the accelerated degradation of the printed robot shell materials after the CCT is 49.18 MPa (8 weeks of salt spray treatment), which reflects the effect of the remaining photocuring agent mentioned above. Generally, the long-term exposure of robot shell materials in outdoor environments neither damages the resin nor increases its reliability in applications such as robot shells. These results demonstrate that the printed robot shell material is suitable for application in open water environments under sunlight irradiation.

### 3.2 Surface Energies of Printed Robot Shell Materials for Weathering Resistance

In addition to the mechanical fracture strength, the surface energy is an important parameter that affects the robot shell material characteristics and is influenced by the weathering effect. Weathering may affect the surface wettability and adhesion characteristics of robot shell materials. The surface energy of a polymeric material primarily depends on the presence of functional groups on its surface, which determine its chemical composition. During the weathering process, polymers can undergo reactions induced by factors such as sunlight irradiation, reaction with oxygen, and humidity, leading to changes in the surface properties.^25^ Specifically, the polymer surface may undergo oxidation, cross-linking and degradation reactions.

Contact angle and surface tension measurements were performed to assess the surface conditions and optical clarity after three months of immersion in lake water. Figure S2 shows the contact angles, which range between 61.4° and 70.1°, indicating slight hydrophilicity. To evaluate whether surface contamination could affect light transmittance during robotic operations, a field test was conducted, and the results are shown in Figure S3. Polished, arc-shaped samples were exposed to two environments: dry soil and mud with a relatively high-water content (1:2 water-to-soil weight ratio). The extent of surface contamination was then observed. Previous studies have suggested that surface contamination is influenced by both surface energy and roughness.^26^ The polishing process effectively reduces the roughness and minimizes dust absorption in dry soil, with minimal impact on image detection. However, in a muddy environment, the hydrophilic nature of the surface affected transparency to some degree. Nonetheless, the transparent appearance of the samples was restored within 5 s of rinsing with tap water. These findings demonstrate that polished, curved spherical shells exhibit fundamental self-cleaning properties, even under conditions of slight hydrophilicity.

To accurately evaluate the surface conditions, the surface energy was calculated using the Owens–Wendt–Rabel–Kaelble (OWRK) method^27^ with two solvents, i.e., water and diiodomethane. This method considers the polarity and dispersion of the solvents to calculate the surface energy. The polar part of one solvent must be greater than zero. Equation 1 describes the relationship between the surface energy and the root-mean-square of each of the two parts.
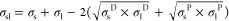
1

**Figure 3:**
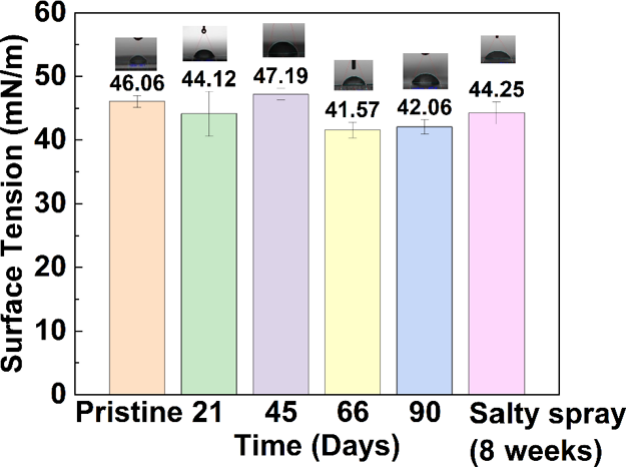
Surface tension values of the printed robot shell materials in the bamboo lake after three months of outdoor exposure (*n* = 5).

As shown in Figure 3, the surface energy of the pristine sample is 46.06 mN/m, with a standard deviation of 0.90 mN/m. As the sample (*n* = 5) immersion time increased, the surface energy values were 44.12 ± 3.51, 47.19 ± 0.89, 41.57 ± 1.23, and 42.06 ± 1.11 mN/m at 21, 45, 66, and 90 d, respectively. The surface energy variations between the samples immersed in lake water for different numbers of days are less than 10%. As discussed previously with regard to the mechanical strength of a printed robot shell material, the surface energy variation may be due to the photocuring agent remaining in the resin after the printing process. However, the obtained surface energy for the accelerated degradation of printing materials after the CCT is approximately 44.25 mN/m, which is very close to that of the pristine sample. Based on the observations of mechanical strength and surface energy, long-term exposure of a robot shell material in an outdoor environment does not damage the resin, and the results support the reliability of this resin in future use as a robot shell material. These results demonstrate that the printed material is suitable for application in an open-water environment under long-term sunlight irradiation.

### 3.3 UV–Vis–Near-IR Diagram of Printed Robot Shell Materials

For robots used for land or water purposes, real-time optical or image sensors inside the spherical robot, such as cameras and laser imaging, detection, and ranging (LiDAR) systems, can be used for autonomous driving or other specific applications.^28−30^ However, optical image sensors rarely work if they are enclosed in an opaque robot shell. Therefore, a 3D-printed poly(methyl methacrylate) (PMMA) resin with high visible light transmittance is chosen as the robot shell material. UV–visible-near-IR measurements were used to evaluate the optical properties of the resin after various lake water immersion times.

**Figure 4:**
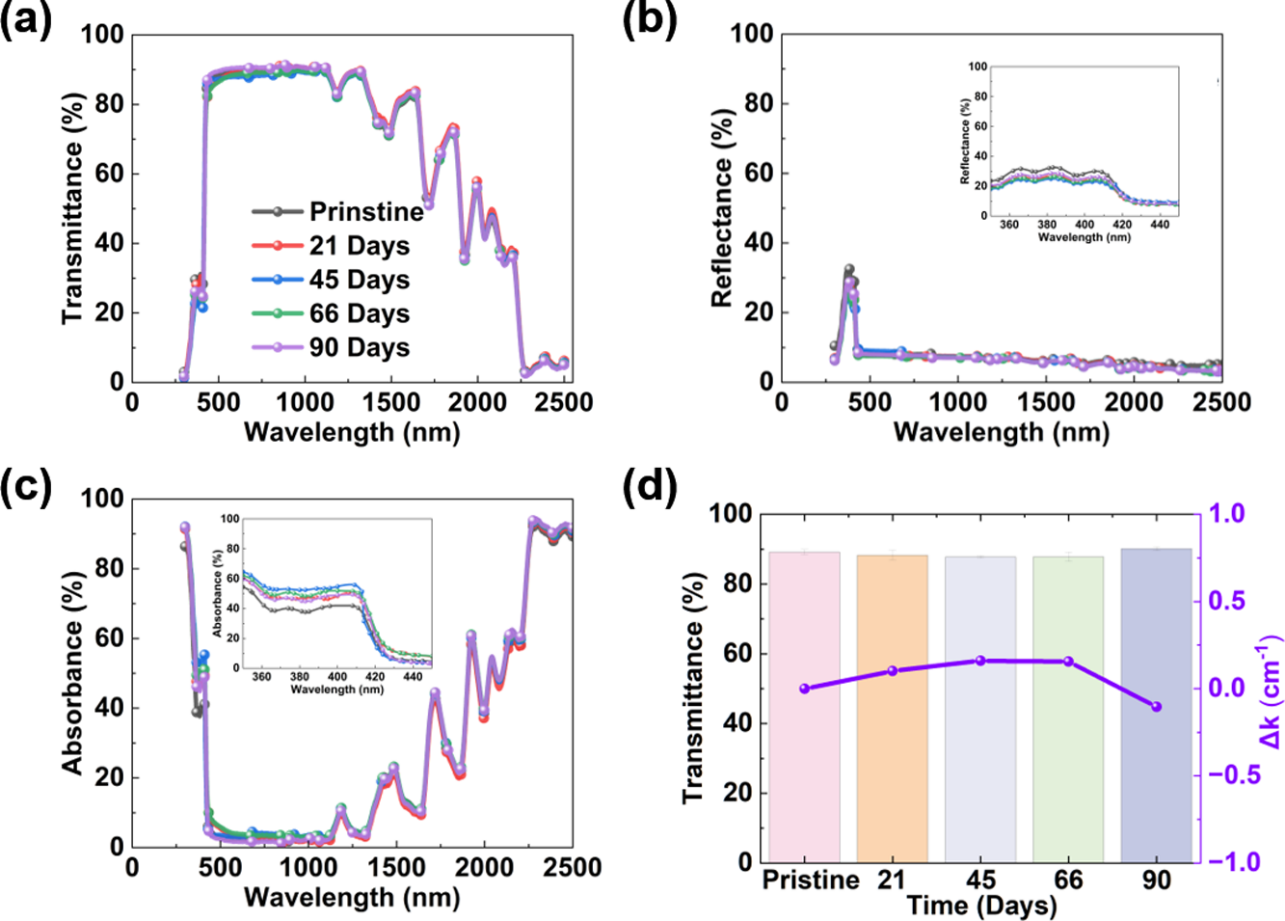
(a) Transmittance, (b) reflectance, (c) absorbance, and (d) Δ*k* in the visible region of the UV–Vis–near-IR spectrum of the printed robot shell materials in the bamboo lake after three months of outdoor exposure.

Figure 4a shows an increase in the transmittance starting at 405 nm, with 90% transmittance at the wavelengths of 450 and 1050 nm. This finding indicates that the robot shell candidate materials have excellent transmittance in the visible region, regardless of whether they are exposed to outdoor environments for three months. The inset in Figure 4b reveals a decrease in the average reflectance between 350 and 450 nm from 32 to 25% after 21 d of immersion. The reflectance of the material subsequently remains constant at 21% after 45 and 66 d of immersion before increasing again to 26% after 90 days of immersion. Interestingly, this trend is similar to the trend observed in the mechanical strength analysis. Furthermore, the results suggest that the impact of the photoinitiator decreases between 66 and 90 d of outdoor exposure. Figure 4c shows typical absorption peaks of the printed robot shell material at 300 and 405 nm, which are attributed to the π → π* transitions of the conjugated backbone.^29^ As the wavelength increases from 300 to 400 nm, the absorbance decreases until it reaches a minimum value. The absorbance then begins to increase again in the visible spectral region between 400 and 450 nm due to the occurrence of transitions involving polymer side groups.

The Beer–Lambert law^30^ is used to assess the weathering durability of 3D-printed robotic shell materials:
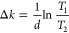
2

In eq 2, Δ*k* is the change in the absorption coefficient, *d* is the sample thickness, and *T*_1_ and *T*_2_ are the transmittances of the printed robot shell material obtained from the visible region recorded before and after three months of outdoor weather aging, respectively. Even though Δ*k* represents the difference in the linear absorption coefficient before and after the exposure weathering test, the magnitudes of Δ*k* shown in Figure 4d are quite close to each other. These results show that the 3D-printed robot shell material behaves as a weathering-resistant material after three months of immersion in outdoor lake water.

### 3.4 Finite Element Simulation for Multifunctional Robot Spherical Shells

**Figure 5:**
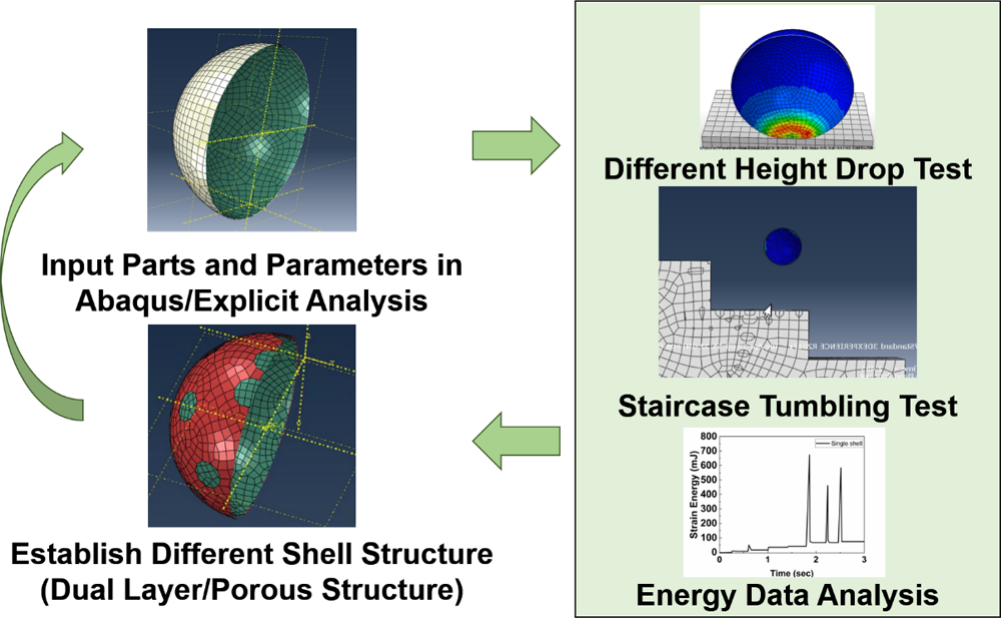
Process flow of the mechanical simulation of printed robot shell materials.

**Figure 6:**
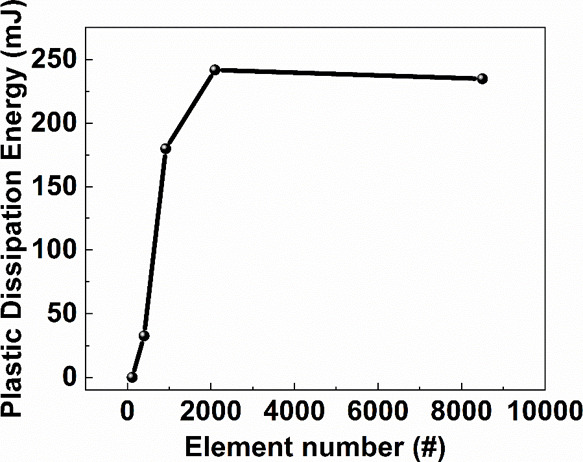
Plastic dissipation characteristics of different mesh layers in a one-meter drop test simulation for printed robot shell materials.

In the field of mechanical simulation, FEA is a powerful tool for solving problems that involve parts that interact with and contact each other.^31^ To achieve high simulation accuracy and efficiency, it is essential to consider various factors, such as appropriate refinement, boundary condition definitions and contact properties. First, the relationship between the residual stress and mesh size is determined. Then, the boundary conditions of the parts are obtained, and the friction coefficient is included in the subsequent actual field simulation (stair simulation) to make the whole simulation realistic. Figure 5 shows the flowchart for the mechanical simulation of different shell parameters in the protection layer. There are two broad categories of approaches for solving contact problems.^32^ Analytical/semianalytical models have low computational costs but may not be as accurate as finite element methods. By contrast, the finite element method is accurate but expensive and requires proper definitions of boundary conditions and meshing. Choosing appropriate mesh partitioning parameters can increase the accuracy and efficiency of simulations. Figure S4 shows a spherical robot shell divided into different numbers of elements. As the mesh size decreases, more elements are generated, improving simulation accuracy. However, overmeshing consumes considerable time and computing resources. The mesh size of the spherical shell was determined by performing impact simulations on a 24 cm diameter spherical shell with an internal load of 3.2 kg. Figure 6 shows the influences of different mesh sizes on the plastic energy dissipation in the drop simulation. When the number of elements exceeds 2000 or the mesh size is less than 1 cm, the simulation results become stable. To achieve a balance between accuracy and simulation time, the mesh size and division method are selected for the following stair drop test.

**Figure 7:**
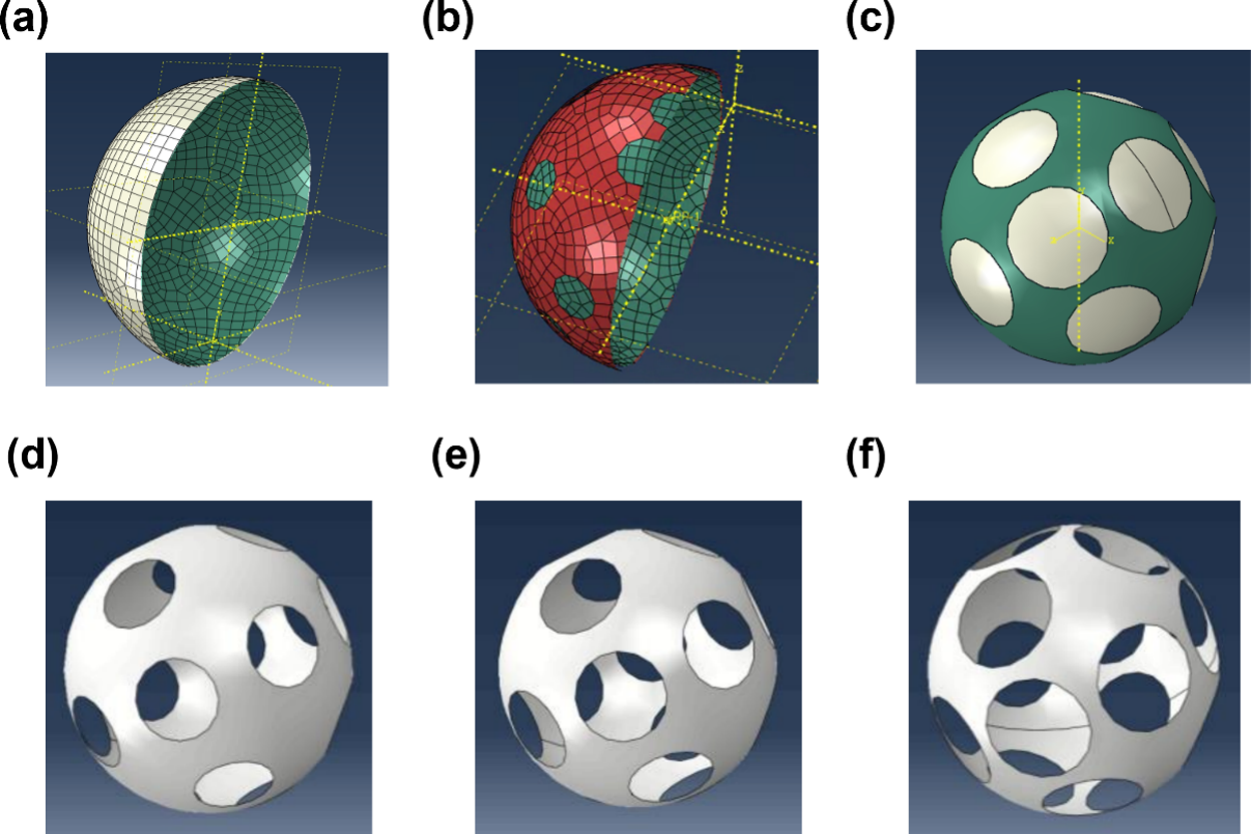
(a) Single-layer, (b) dual-layer, (c) full dual-layer and outer-shell structures with (d) 30%, (e) 40%, and (f) 50% porosities for printed robot shell materials according to the Abaqus simulation.

Next, all the components are assembled in the digital model, and the necessary contact and boundary conditions, such as the friction coefficient and fixed stair parameters, are established. The structures of the outer protective shell and the inner transparent shell are shown in Figure 7, and both shells are divided into elements with the dimensions of approximately 2 × 2 mm. Table 1 shows the energy data for different structures of printed robot shell materials including the single-shell design during the stair drop simulation. After dropping from a height of 1 m, obvious tiny cracks appeared. To increase the mechanical strength, a double-layer spherical shell design is implemented. The inner layer has a thickness of 1 mm, whereas the protective layer has a thickness of 2 mm. The outer layer of the protective shell is made from another printable resin with excellent stretch ability and deformability. To preserve the high optical performance of the inner shell, a porous structure is employed. In the optimization of the porous structure, circular shapes offer higher symmetry and distribute strain evenly than other common designs such as square holes and linear structures. This approach not only reduces material consumption but also ensures that the optical properties of the inner layer are maintained. The mechanical properties of the two materials are summarized in Table S1. The elasticity of the outer layer enables it to effectively resist sudden impact forces and act as an elastomer coating, enabling impact absorption. This arrangement helps absorb a portion of the impact force between the shell and the ground, storing the impact energy in the outer elastic layer and releasing it through multiple bounces. Consequently, the bouncing process prevents the shell material from breaking. Through mechanical simulations, deformation of the inner shell from an energy perspective is observed. The train energy, which is a type of potential energy that is stored in a structural member because of elastic deformation, is an important factor. The strain energy diagram is shown in Figure S5 and reveals a reduction in the residual strain energy from 130 to 22.09 mJ. Furthermore, the inner shell with a 50% porous protective layer does not exhibit any tiny cracks. Interestingly, a higher porosity leads to improved protective performance. Notably, the protective shell possesses a porous structure with *x*–*y*–*z* three-axis symmetry, as shown in Figure 7d–f. Nevertheless, when the porosity exceeds 60%, the spacing between lightweight structures becomes too small to ensure sufficient impact resistance. It is essential to strike a balance between the circumference of each hole in the structure and its mechanical endurance. Owing to the limitations imposed by the printed material and structure design, the highest porosity needed to achieve a lightweight structure is 50%. This result can be attributed to the following factors. The first factor is the low weight of the double-layer spherical robot shell. Through the contribution of high porosity, the impact force can be effectively reduced. The other factor is the presence of large pores, which provide sufficient space for strain release. This design ensures that the impact stress is uniformly distributed across the entire hole circumference. However, a too large size of the hole compromises the strength of the shell, making it unable to withstand multiple impacts. Therefore, according to the simulation, a protective layer for the robot shell with the hole ratio of 50% and an inner layer consisting of an outer shell are required to maintain optical transparency and ensure adequate mechanical protection for future robot operation on land and water.

**Table 1:** Energy Data for Different Structures of Printed Robot Shell Materials during Stair Drop Simulation

	single layer shell	30% porosity shell	40% porosity shell	50% porosity shell
residual strain energy (mJ)	130	97.02	92.01	22.09
damage dissipation (mJ)	1319	77.08	70.99	6.04

Real drop tests were also conducted to verify the mechanical properties and protective capability of the spherical shell. To facilitate the testing process, a scaled-down model of a sphere was fabricated via 3D printing. The model was one-third the size of the original sphere and had an inner sphere diameter of 8 cm. Drop tests were performed at two different heights: 30 cm, which is equivalent to the height of one stair step, and 1 m. Additionally, simulated stair descent was also conducted. Video S1 shows that the single-layer sphere suffered significant damage when it was dropped from a height of 30 cm. However, when a porous outer protective layer was added, the mechanical properties of the spheres were notably enhanced. This led to the preservation of structural integrity during both the 1 m drop and the multistep stair descent tests, as demonstrated in Videos S2 and S3. These results highlight the protective effectiveness of the double-layer spherical shell against impacts, emphasizing its potential in application as a protective shell for spherical robots.

### 3.5 FTIR Spectrum of the 3D Printed Robot Shell for Daytime Radiative Cooling

Daytime radiative cooling is effective in various electronics, reducing energy use and mitigating global warming.^33^ Integration of daytime radiative cooling materials into the design of spherical robotic shells enhances their thermal management capabilities in harsh environments. This approach is particularly useful for applications where traditional cooling methods such as convection or conduction are impractical or insufficient. Previous studies have demonstrated that materials with emittances in the range of 8–13 μm can undergo passive cooling.^34^ The radiative cooling mechanism involves the dissipation of heat in the atmospheric window into space through thermal radiation energy. Outside this range, the radiated energy is suppressed due to the low infrared transmittance of air. Equation 3 for calculating the cooling efficiency is as follows^35,36^:
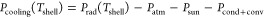
3where *P*_cooling_(*T*_shell_) is the cooling efficiency of the shell material; *T*_shell_ is the surface temperature of the printed sample; and *P*_rad_(*T*_shell_) is the radiative energy emitted from the material surface. *P*_atm_ is the background thermal radiation absorbed by the material from the atmosphere; *P*_sun_ is the solar radiation absorbed by the material; and *P*_cond+conv_ represents the nonradiative heat exchange between the material and the surrounding environment. Further discussion regarding these factors for the calculation of the radiation cooling power can be found in Supplementary Note S1.

**Figure 8:**
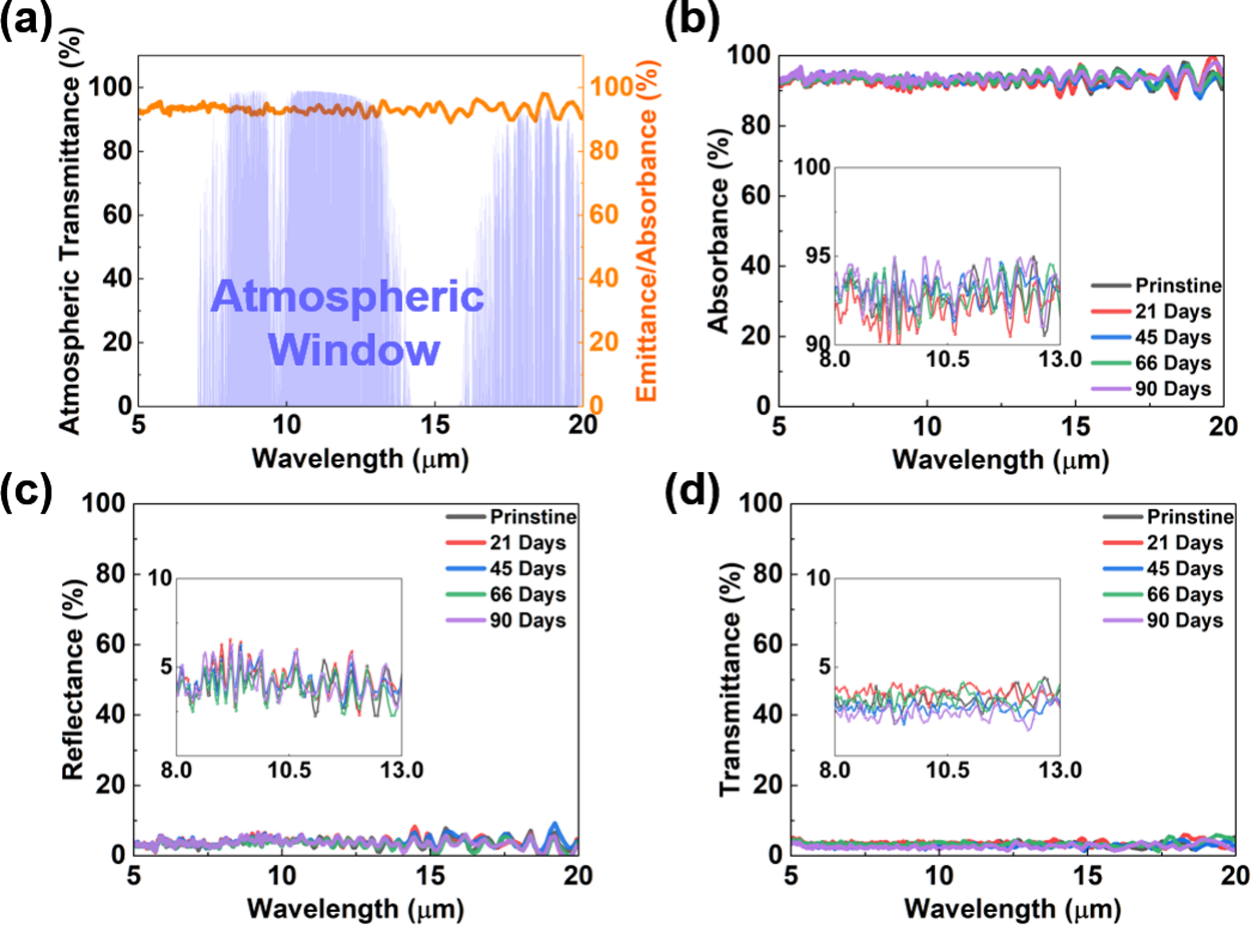
FTIR spectra of the (a) atmospheric window and emittance/absorbance of the pristine printed sample and the (b) absorbance, (c) reflectance and (d) transmittance values of the printed robot shell materials immersed in a bamboo lake for three months.

The results indicate that the high emittance of materials in the 8–13 μm range ensures high cooling efficiency. Hence, optimal performance can be achieved by focusing on the emittance performance in the mid-IR range. Figure 8 shows the obtained FTIR spectra used to determine the transmittance and absorption characteristics of the printed robot shell material in the infrared region. A 3D-printed resin exhibits multiple advantages for use as a passive cooling material, such as being low weight and suitability for customizable design. This material can accommodate small variations in microsphere size and shape, with a negligible impact on the overall performance. Figure 8a shows the high emittance of the printed sample in the first (8–13 μm) and second (16–25 μm) atmospheric windows. The high emittance depends on the good absorption property (Figure 8b) of the printed robot shell material. The blue shaded area represents the normalized intensity of air mass 1.5 with a water vapor column of 1.0 mm. The emittance of eq 4 is calculated using Kirchhoff’s law,^37^ which states that the emittance of a material in thermal equilibrium is as follows:
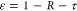
4where ε is the emittance, *R* is the reflectance, and τ is the transmittance of the printed robot shell material. In the 8–13 μm atmospheric window, the reflectance in Figure 8c is low at approximately 4–6%, whereas the transmittance in Figure 8d is also low at approximately 2.5–4%. This finding indicates the high absorbance and high emittance in heat equilibrium. These properties of robot shell materials endow them with great potential for use in daytime radiative cooling applications. After a 3-month exposure period, the effective emissivity (ε_eff_) across the entire MIR range (5–20 μm) changes from 0.876 to 0.884. This finding demonstrates outstanding weather resistance. Furthermore, regardless of outdoor exposure, the absorbance at the atmospheric window (8–13 μm) consistently exceeded 90%. The daytime radiative cooling mechanism can help reduce the temperature in this multifunctional spherical shell and enable computer and optical electronics to work for relatively long periods. A previous study illustrated that radiative cooling materials with a high effective emissivity (ε_eff_ ≈ 0.88) can lower the temperature of a mobile phone by approximately 5 °C.^35^ However, the printed material in this study primarily exhibits thermal emission in the MIR region. Its 2 mm thickness and inherent material properties result in increased near-infrared (NIR) absorption and high transparency in the visible region, both of which can negatively impact the overall cooling performance. Nevertheless, due to its high emissivity the material contributes to some degree of cooling. Moreover, the stable performance of the martial makes it highly advantageous for long-term outdoor applications, particularly for spherical robots that accumulate a significant amount of internal waste heat and require efficient radiative cooling.

## 4 Conclusions

This study investigated the mechanical and weatherability characteristics of a spherical robot shell fabricated using SLA 3D-printed resin. The experimental results demonstrated that the shell maintained excellent mechanical strength and optical performance even after three months of outdoor exposure in a lake environment. Notably, the tensile strength of the shell material increased slightly during this period, with a variation of less than 5%. This improvement can be attributed to either minor variations in the 3D-printed structure or cross-linking recombination induced by sunlight exposure. Additionally, no significant changes were observed in the surface energy, suggesting that the surface properties of the material remained unaffected. The spectrum in the visible light region revealed that the transmittance was greater than 85%. Mechanical simulations demonstrated that the double-layered structure effectively reduced the residual strain energy, with the structure with 50% porosity providing optimal protection by achieving an 80% reduction in the residual strain energy compared with that of the pristine single-layer shell. Based on the above observations, the printed robot shell showed perfect stability during three months of outdoor tasks. Moreover, FTIR spectral analysis revealed the radiative cooling capability of the inner spherical shell. Furthermore, a high emissivity in the mid-infrared region was observed for the printed inner shell, and the corresponding atmospheric light window of the FTIR spectrum facilitated radiation cooling. The emissivity performance of the robot shell material was retained after immersion in a lake for three months. The optical properties may endow this material with high potential for its future practical application in the passive cooling of robot systems. Future studies can explore advanced topology optimization techniques to improve the mechanical properties of shells. The implementation of designs such as multilayer or hybrid porous structures may further improve strength-to-weight ratios while maintaining protective functionality. These developments can expand the applications of the protective layer in diverse engineering fields.

## Supporting Information Available

Properties of different materials during stair drop simulation (Table S1); (a) top view and (b) cross-sectional view of the dog bone sample used for the tensile strength test (Figure S1); contact angle values for printed robot shell materials: (a) pristine, (b) 21 d, (c) 45 d, (d) 66 d, (e) 90 d of outdoor exposure and (f) 8 w of salt spray treatment. (Figure S2); appearance of the polished and arc-shaped sample subjected to different conditions. (a) Initial state of the sample. Sample after being rolled in (b) dry soil and (c) mud (water-to-soil weight ratio of 1:2) for 5 min. Transparency restored after rinsing with tap water following exposure to (d) dry soil and (e) mud (water-to-soil weight ratio of 1:2). (f) Final appearance observed under natural light. (Figure S3); illustrations of a spherical robot shell divided into different numbers of elements: (a) 115, (b) 400, (c) 920, (d) 2100, and (e) 8500 (Figure S4); and strain energies of various double-layer spherical robot shells during a stair drop simulation (Figure S5) (PDF)
Single-layer sphere without or with a shell dropping from a height of 30 cm (MOV)
Single-layer sphere with shell protection dropping from a height of 1 m (MOV)
Single-layer sphere with shell protection dropping from multistep stair (MOV)

## Supplementary Material

ao4c09954_si_001.pdf

ao4c09954_si_002.mov

ao4c09954_si_003.mov

ao4c09954_si_004.mov
