# Supplementary material for: Design of a Multifunctional Resin-Based Outdoor Spherical Robot Shell for Ultrahigh Visible to Near-Infrared Transmittance and Mid-Infrared Radiative Cooling
Source: ACS Omega. 2025 Jan 11;10(3):3080–9. doi: 10.1021/acsomega.4c09954 (PMC11780446; doi:10.1021/acsomega.4c09954)
Supplement: Supplementary file 1 — ao4c09954_si_001.pdf [file ao4c09954_si_001.pdf]

Supporting Information

Design of a Multifunctional Resin-based Outdoor Spherical Robot Shell for Ultrahigh Visible to Near-Infrared Transmittance and Mid-Infrared Radiative Cooling

Wei-Lin Wu[1], Shang Yu Tsai[1], Yu-Chieh Lo[1], Hsueh-Cheng Wang[2], Hsuen-Li Chen[3], Dehui Wan[4] and Fu-Hsiang Ko[*1]*

[1]Department of Materials Science and Engineering, National Yang Ming Chiao Tung University, No. 1001, University Road, Hsinchu 30010, Taiwan

[2]Department of Electronics and Electrical Engineering, National Yang Ming Chiao Tung University, No. 1001, University Road, Hsinchu 30010, Taiwan

[3]Department of Materials Science and Engineering, National Taiwan University, No. 1, Sec. 4, Roosevelt Road, Taipei 10617, Taiwan

[4]Institute of Biomedical Engineering, National Tsing Hua University, No.101, Sec. 2, Kuang Fu Road, Hsinchu 30013, Taiwan

Corresponding author: fhko@nycu.edu.tw

Supplementary Note S1

Calculation of the radiation cooling power

$$P_{cooling}(T_{shell}) = P_{rad}(T_{shell}) - P_{atm} - P_{sun} - P_{cond+conv} \qquad (1)$$

where T_{shell} is the surface temperature of the printed sample and $P_{rad}(T_{shell})$ is the radiative energy emitted from the material surface that can be calculated via the following equation:

$$P_{rad}(T_{shell}) = \pi \int_0^{\frac{\pi}{2}} \int_0^{\infty} \varepsilon_r(\lambda,\theta)\ I_{BB}(\lambda,T_{shell}) \sin 2\theta \, \mathrm{d}\lambda d\theta \qquad (2)$$

$$I_{BB}(\lambda,T_{shell}) = \frac{2hc^2}{\lambda^5} \ \frac{1}{e^{\frac{hc}{\lambda k_B T}} - 1} \qquad (3)$$

where $\varepsilon_r(\lambda,\theta)$ is the emissivity of the 3D-printed sample in the mid-infrared (MIR) range; $I_{BB}(\lambda, T_{shell})$ is blackbody radiation, representing the maximum possible radiative energy at that temperature; h is the Planck constant; c is the velocity of light in vacuum; and k_B is Boltzmann's constant. The integral expression of $P_{rad}(T_{shell})$ shows the total radiative power at the surface of the material, which signifies the amount of heat released through radiation and determines the material's cooling capability.

$$P_{atm} = \pi \int_0^{\frac{\pi}{2}} \int_0^{\infty} \alpha_r(\lambda,\theta)\ I_s(\lambda,\theta,T_{shell}) \sin 2\theta \, \mathrm{d}\lambda d\theta \qquad (4)$$

where $\alpha_r(\lambda,\theta)$ is the absorbance of the 3D-printed sample, which is equal to the emittance of the 3D-printed sample $\varepsilon_r(\lambda,\theta)$ according to Kirchhoff's law. $I_s(\lambda,\theta,T_{shell})$ is the solar radiation energy.

P_{atm} is the background thermal radiation absorbed by the material from the atmosphere. This radiation is generally low, particularly on clear nights when there is minimal radiation energy at the atmospheric window, thereby enhancing the cooling effect.

$$P_{sun} = \int_0^\infty \alpha_r(\lambda,\theta_{sun})\ I_{AM1.5}(\lambda)\mathrm{d}\lambda \qquad (5)$$

where $I_{AM1.5}(\lambda)$ is the reference direct normal spectral irradiance ASTM 1.5G.

P_{sun} is the solar radiation absorbed by the material. To achieve high reflectivity and minimize heat input, effective cooling materials should minimize absorption in this area.

$$P_{cond+conv} = Ah_c(T_{amb} - T_{shell}) \qquad (6)$$

$P_{cond+conv}$ represents nonradiative heat exchange between the material and the surrounding environment. Energy transfer is influenced by factors such as the environmental temperature, material thermal conductivity, and air flow. Efficient passive cooling can be achieved by appropriately designing the optical and thermal properties of materials to maximize $P_{rad}(T_{shell})$ while minimizing P_{sun} and $P_{cond+conv}$.

$$\varepsilon_{eff} = \frac{\int_{2.5\ \mu m}^{25\ \mu m} I_{BB}(\lambda,T) \times \varepsilon_r(\lambda,T) d\lambda}{\int_{2.5\ \mu m}^{25\ \mu m} I_{BB}(\lambda,T) d\lambda} \qquad (7)$$

where ε_{eff} is the effective emissivity; T is set at 300 K; and ε_{eff} is the overall radiation efficiency of the material under specific conditions, considering the impact of various factors, including wavelength, angle, and temperature.

Table S1. Properties of different materials during stair drop simulation.

	Material	Young's modulus (GPa)	Poisson's ratio	Elongation at break (%)	Tensile strength (MPa)
Stair	Aluminum	200	0.3	-	200000
Inner shell	PMMA (Clear resin)	1.9	0.35	6%	50
Outer shell (Protective shell)	PMMA (Durable resin)	0.45	0.35	55%	31.8

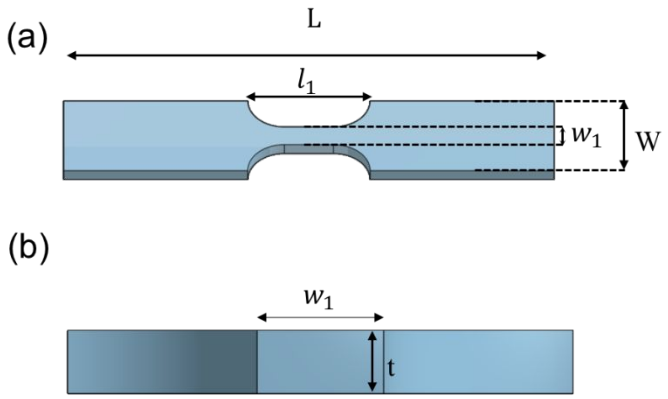

Figure S1. (a) Top view and (b) cross-sectional view of the dog bone sample used for the tensile strength test.

Total length:$L = 40\ mm$

Length of the narrow parallel part:$l_1 = 10\ mm$

Sample width:$W = 8\ mm$

Sample width in the measuring length range:$w_1 = 2\ mm$

Thickness:$t = 1\ mm$

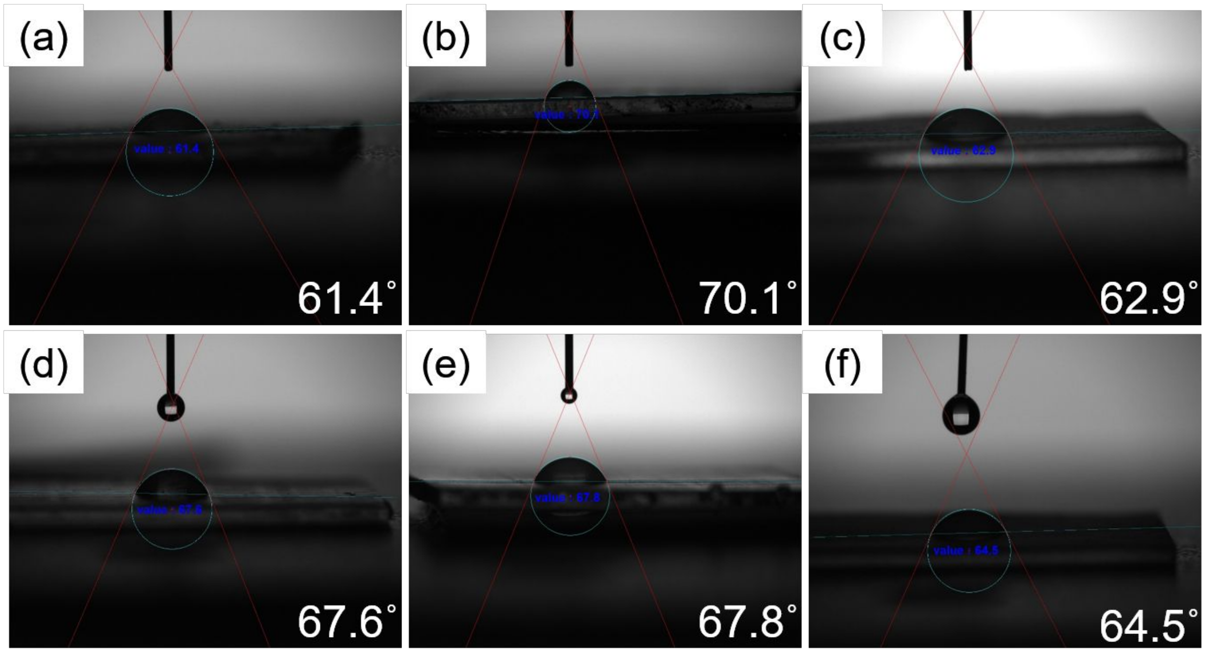

Figure S2. Contact angle values for printed robot shell materials: (a) pristine, (b) 21 d, (c) 45 d, (d) 66 d, (e) 90 d of outdoor exposure and (f) 8 w of salt spray treatment.

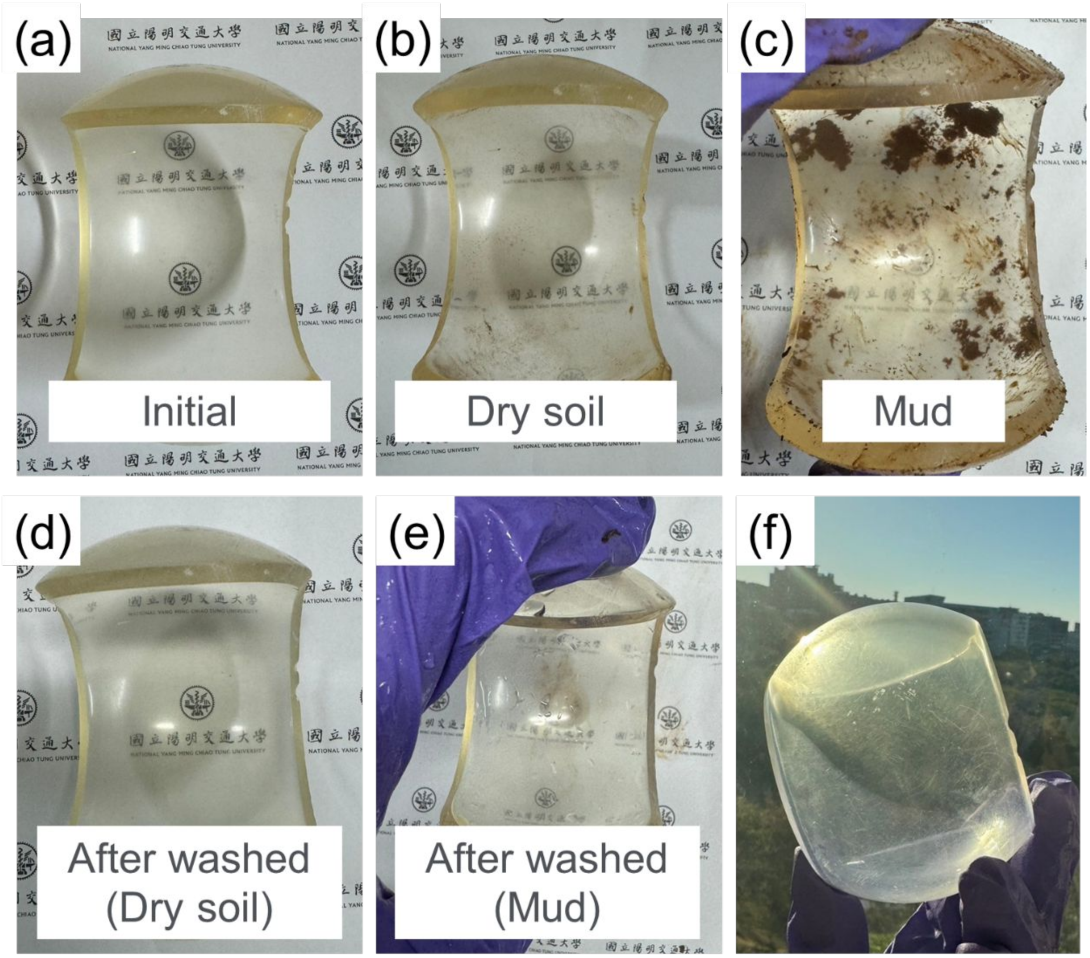

Figure S3. The appearance of the polished and arc-shaped sample subjected to different conditions. (a) Initial state of the sample. Sample after being rolled in (b) dry soil and (c) mud (water-to-soil weight ratio of 1:2) for 5 minutes. Transparency restored after rinsing with tap water following exposure to (d) dry soil and (e) mud (water-to-soil weight ratio of 1:2). (f) Final appearance observed under natural light.

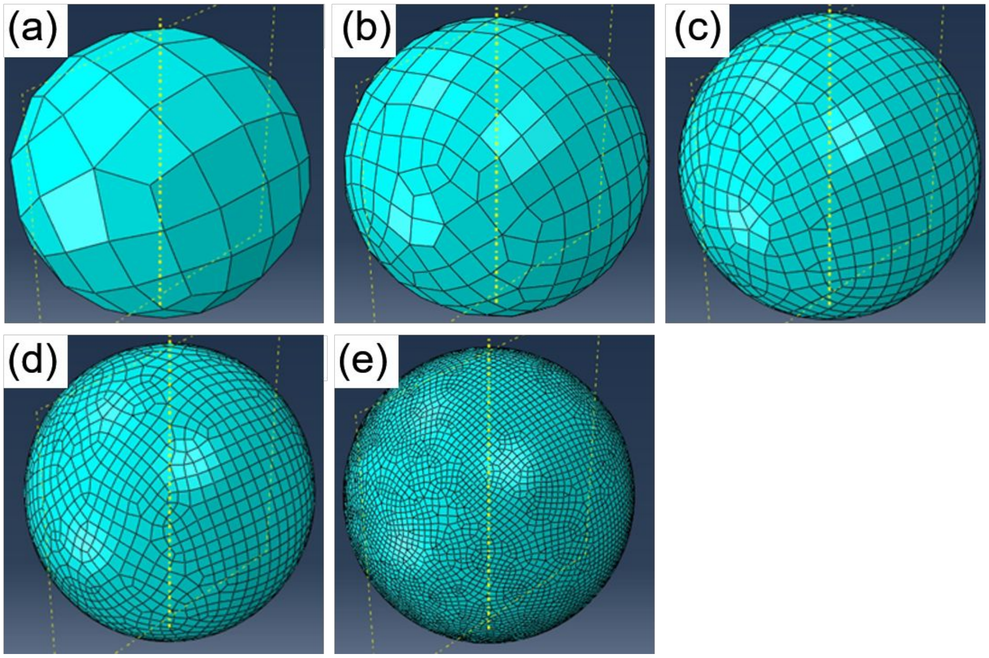

Figure S4. Illustrations of a spherical robot shell divided into different numbers of elements: (a) 115, (b) 400, (c) 920, (d) 2100, and (e) 8500.

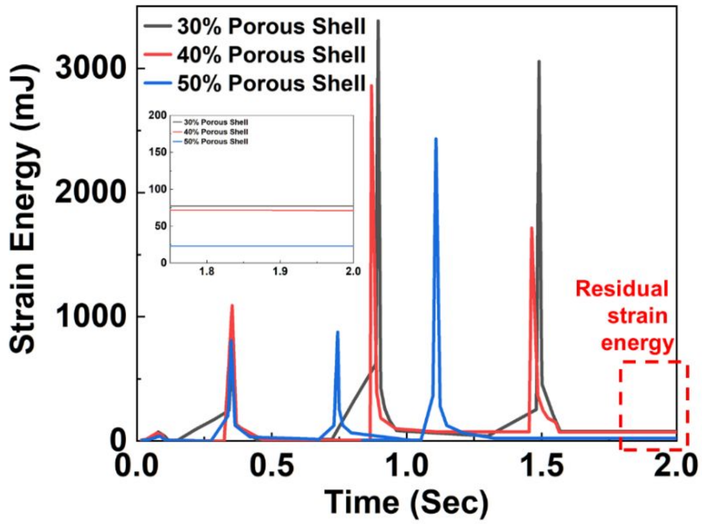

Figure S5. Strain energies of various double-layer spherical robot shells during a stair drop simulation.
